# Opium in Metro-Peritonitis

**Published:** 1871-02

**Authors:** Theodore Griffin

**Affiliations:** 529 State street, Chicago


### Selection, and Comments.

*Opium in Metro-Peritonitis.* By Theodore Griffin, M.D., 529 State street, Chicago.

Appreciating the fact that it is only by recording and giving to our brethren the results of carefully-observed experience in the treatment of disease, that true and reliable progress is made in therapeutics, I offer for consideration my record of the following case of metro-peritonitis, treated with opium.

I present it briefly and without comment, as a link added to the chain of evidence in favor of the use of opium in this fearful disease. I was led to the employment of large doses by the plain indications to be fulfilled, which I know the physiological effect of opium would meet, bearing in mind this fact, which I think should become an axiom: *if indications are scrupulously met in treatment, no drug poisoning, with its disagreeable and dangerous perturbations, will occur, howsoever large the dose of the drug may be.* It is a lamentable fact that indications are often *more than met*, as occurred at the close of this case through the ignorance of the nurse. * * *

On the 16th day of June, 1870, I attended Mrs. —— in labor. She says that after previous labors, numerous clots of blood were always expelled from the uterus; after this labor, none were expelled.

On the 17th, she complained of severe pain over the region of the uterus, especially upon the right side, describing it as sharp and lancinating. She had, on this day, a severe chill; her tongue was covered with a white fur; her bowels were constipated; her pulse 90, and bounding. She had, also, headache and aching of the limbs. These symptoms, however, yielded to a little mild treatment, and on the evening of 19th, she felt, as she expressed it, and as her symptoms indicated,—" quite well."

On the morning of the 20th, at 3 A. M., more lancinating pains began in the uterus and over the abdominal region, accompanied by all the initial symptoms of metro-peritonitis. I immediately directed that she have *one gr. of opium every hour.* The pain continued unabated until 9 o'clock P. M. At this time her appearance indicated great suffering; her nervous system was disturbed and agitated; her tongue coated with a dirty black coat; there was nausea and vomiting, with fetid and dark-colored discharges from the bowels; the slightest touch or motion produced the most intense pain; the abdomen was distended to its size immediately previous to confinement; her pulse was 110. I now gave *three grs. of opium* at one dose, and ordered *one gr. to be given every half-hour.* At 8 o'clock A. M. (21st) her pulse was 94, her tongue was still black, but somewhat improved; no more vomiting nor fecal discharges since my last visit; the pain was yet severe. I now gave *four grs. of opium at one dose*, and ordered *two grs. of opium to be given every half-hour.* At 11½ o'clock A. M., her pulse was 100; the pain was still unabated; no signs of opium poisoning. She has passed no urine since 12 o'clock last night; the mammary secretion was now gone, and pus discharged continuously from the vagina. I ordered *five grs. of opium to be given every hour.*

At 4½ o'clock P. M., (21st), nervous symptoms were better, she had less pain and rested comparatively easy; some subsultus tendinum. I now directed *three grs. of opium to be given every half-hour*, and drew her urine with the catheter. At 10 o'clock P. M. she rested well, there was now no subsultus; she had now some inclination to sleep; her pupils were natural and her pulse 100; continued the opium, three grs. every half-hour. At 11 o'clock P. M she slept for the first time since her illness began; at this time I left her for the night, directing the nurse to discontinue the opium if she awoke without pain, and with an inclination to sleep. At 2 o'clock A. M., (22nd), she awoke, bright, intelligent, and free from pain, and, as she told the nurse, *feeling well* The nurse, however, continued the opium as before, *three grs.* every half-hour. At 5 o'clock A M. I was called to her in great haste, and found her narcotized, breathing stertoriously with contracted pupils; these alarming symptoms, resulting from the *indications beinr more than met*, quickly disappeared with appropriate treatment. She soon became conscious, convalescence being established from that moment. There remained some tenderness on pressure over the region of the uterus, but the distention of the abdomen had entirely disappeared with all the symptoms pertaining to the digestive and nervous systems; her pulse was natural, and her restoration to perfect health was rapidly completed.

The amount of opium given is presented in the following summary:

From 3 o'clock A. M., 20th, to 9 P. M., 18 hours, 18 grs.; from 9 o'clock P. M., 20th, to 8 o'clock A. M., 21st, 11 hours, 23 grs.; from 8 o'clock A. M., 21st, to 11½ A. M , 3½ hours, 18 grs.; from 11½ o'clock A. M., 21st, to 4½ o'clock P. M , 5 hours, 25 grs.; from 4½ o'clock P. M., 21st, to 10 o'clock, P. M., 5½ hours, 33 grs.; from 10 o'clock P. M., to 11 o'clock P. M., 1 hour, 6 grs. Total—44 hours, 123 grains.

At 2 o'clock A. M., 22nd, 6 grs. The last 6 grains produced the narcotism. No other drug than opium was administered during the entire course of the disease.

We transfer the above communication to the columns of the JOURNAL *verbatim*, with the intent to direct professional attention to a certain reckless style of journalism, too much in vogue with those having the editorial control of medical periodicals, which permits the publication of articles calculated to mislead the unwary, and possibly to occasion disastrous results. Editors of medical journals may officially disclaim all responsibility for the character of communications published in their columns, and yet can never escape the consequences of a certain degree of reflected authority which their admission confers. [We have ourselves suffered from this *reflex* hypothesis. *A.*] Published as an illustration of the degree to which the human system can be educated into the frightful habit of opium-eating, its propriety would be questionable, in view of the rapid spread of this baneful practice, but when given to the professional world as an example of the employment of the drug in legitimate therapeutics, with even the remote possibility of finding an imitator, it should not be permitted to pass by without a recorded protest.

Its prefatory paragraph permits the inference, that its author presents the above as an example of *carefully observed* experience in the treatment of disease—an example which we trust will find but few, if any, imitators. The careful observation in the case being limited to the detail of a few of the more obvious symptoms, couched in language so vague and indefinite as to have little or no value for the purpose of comparison and analysis.

In the next paragraph we are told that the writer "was led to the employment of large doses (of opium) by the plain indications to be fulfilled"; and he then presents an array of symptoms, such as, "bowels constipated," "pulse 90 and bounding," etc., which, according to the teachings of modern physiology, directly contra-indicate its use.

In regard to the italicized passage in the second paragraph, which the writer thinks should become an axiom, it may be said, that axioms, being self-evident propositions, are such essentially, and cannot become such; and moreover, that this proposed axiom involves both a verbal and a therapeutic paradox, the second and third terms each containing a possible contradiction of the first.

That it is a lamentable fact, that "*indications are often more than met*," "'Tis true 'tis pity, and pity 'tis 'tis true"!

In the next paragraph are detailed "symptoms which yielded to a little mild treatment," whether embracing heroic doses of opium we are not informed, but sufficiently efficacious to remove all the symptoms in forty-eight hours; which (symptoms), after the relapse on the following day, were not even slightly ameliorated by eighteen grains of opium in as many hours. The result of this experiment, we should suppose, would have been sufficient to skake the faith of Coleridge or De Quincy; but although the patient was evidently much worse, and her "*nervous system much disturbed and agitated*," the dose was rather more than doubled. And again, after the administration of 23 grains in 11 hours, and a considerable improvement in the patient's condition, this last dose was nearly tripled, viz., 18 grains in 3½ hours; and still again, at 11½ A. M., there being "no signs of opium poisoning,"(?) although no urine had been passed for 11½ hours! and "the mammary secretion was gone"! 25 grains were given within five hours! After which, although the "nervous symptoms were better," "with some subsultus tendinum"! the dose was again increased to 3 grains every half hour, 33 grains in 5½ hours, and the urine was drawn with a catheter, after an interval of repose, for that function, of 16 hours; still the opium is pushed with unremitting assiduity, and in the next hour 6 grains are given, making a grand total of 123 grains in 44 hours.

Now mark the change in the tolerance of the drug by the patient: at 2 A. M., 3 grains every half hour were given, until 6 grains had been taken, which "*last six grains produced the narcotism, the indications being more than met*"! It would seem so indeed, and how outrageous the conduct of a nurse, who should administer a quantity, in excess of her instructions, equal to 4.88 per cent.

Here is presented a persistent, though hardly consistent, faith in the efficacy of a drug, for throughout all the fluctuations of the case, the remedy is applied in increasing doses. Was she better? more opium! Was she unchanged? more opium!! Was she worse? still more opium!!!

The analysis of the case suggests the following subjects of inquiry:

1. The propriety of administering a cardiac stimulant and arterial sedative, when the reciprocating equilibrium between these two portions of the circulatory system was already disturbed by the increased activity of the heart.

2. The total failure of the opium to produce any of its constitutional reactions, aside from "disturbance and agitation of the nervous system," "subsultus tendinum," and arrest of the renal and "mammary secretions," which the author evidently excludes from that category.

3. The persistence in the administration of a drug which had so entirely failed, not only to meet the indications, but even to manifest any of its usual effects.

To the first, we must offer our protest in the name of physiology, whose laws it violates.

Of the second, there are several possible explanations.

1st. That the medicine was not taken, which, of course is inadmissible, as it involves the good faith of the physician, or nurse, although the latter is not above suspicion—witness the six grains in excess of orders.

2d. That the drug was worthless, which impugning the integrity of the druggist must be rejected.

3d. That the medicine was pure, and was taken, but was passed unchanged through the intestinal canal, and deposited in the close stool, which has happened before. And—

4th (which we believe to be the true solution of the mystery), The woman was an opium-eater already!

Should this be true, a statement to that effect would have been valuable in elucidating a very "strange story."

Apropos of the third subject of inquiry, we can only suggest for its solution, faith, which, although it may remove mountains, will scarcely contract dilated arteries, arrest subsultus tendinum, or restore functional energy to the kidneys or mammæ, even with the aid of more opium!

We expect soon to hear of administering Hydrocyanic Acid in fifteen drop doses.

W. H.

---

Dr. Guyot gives from 30 to 60 grains of phosphate of lime in profuse perspiration, and especially in night sweats incident upon phthisis pulmonalis. He mixes the phosphate with loaf sugar, and requires the patient to take a pinch of the powder frequently through the day.—*Revue Therapeutique.*

